# Diseases of the Bladder and Prostate

**Published:** 1890-11-01

**Authors:** 


### THE LONDON POST-GRADUATE COURSE AT THE PADDINGTON INFIRMARY.

### DISEASES OF THE BLADDER AND PROSTATE.

Mr. Reginald Harrison in his lecture first thanked Dr. Savill and his colleagues for their kindness in permitting him to lecture at the Paddington Infirmary, and then proceeded to say that he had come rather as a demonstrator than as a lecturer. The resources of the infirmary being very considerable, he had collected a series of cases of sufficient interest for consideration.

Case I. was that of a man, 54 years of age, who was recovering from a severe form of stricture. The patient was admitted on September 24th. The age of the man suggested at once that the case was probably not one of prostatic disease, but of organic urethral stricture. The man was, in fact, suffering from incontinence of urine. In these cases the bladder can only get over the resistance of a tight stricture by keeping the urine constantly on the dribble. Upon examination it was found that there was a very tight stricture in the usual position, namely, in the membranous part of the urethra, and no instrument could be got through. With regard to the choice of instruments, one or other of the soft kinds was preferable. On examination, the urine was found to be of a specific gravity of 1,018, acid, clear, and that there was no albumen. The presumption from this was that there was no kidney mischief to speak of. If it is found that the urine presents a very low specific gravity, or is otherwise abnormal, it is unwise to hastily submit the patient to any severe operation. In this case the man was given one or two days' rest in bed; he having no urgent symptom when admitted, there was no cause for immediate interference; his bladder was not much distended, and he was in no pain. At the end of two days' rest a Number One catheter was passed without difficulty through the stricture into the bladder. The instrument was then tied in and left, in other words, the case was submitted to "continuous dilatation." In these cases it matters very little if a catheter or a bougie be passed and tied in, as the patient may pass his water by the sides of the instrument. The further progress of the case was that on October 4th No. 1 was tied in, on October 5th No. 3, and on October 9th Nos. 4 and 5, and at this stage the man began to micturate in a fair stream. As a rule in a patient so circumstanced, it is the duty of the medical man to impress upon him the great importance of passing an instrument regularly, even long after all symptoms have ceased, and he should be made to understand that he must continue to do this for the rest of his life, once a fortnight, or perhaps only once a month, as the case may be. If, as sometimes happens with these strictures, it is not possible even to pass a No. 1, it is not wise to, as it were, shoot blindly at the stricture. In these cases often the difficulty may be overcome by using a so-called "tunnelled sound," invented by Professor Gouley, of New York. This consists of a small grooved sound, which is passed to the seat of the stricture, and then a very fine whalebone bristle is pushed along the groove, and thus guided into the opening of the stricture. Again, sometimes Banks' filiform bougies are of great service; these, which are exceedingly fine at the point, gradually become larger towards the other end, and so, if once passed through the obstruction, with a little manœuvring a complete dilatation may be performed at one sitting, or at least considerable progress made. The answer to the question as to which instrument is best in any particular case, is, that the one is best which goes in easiest; if a soft elastic one readily passes, this is best; if, on the other hand, it is found that a metal one is more readily got in, then that is the best.

The second case was that of a man who had left the hospital just over a year previously, with a fully dilated stricture, and was told to pass an instrument regularly, but had omitted to do so, and thus in a little over twelve months he had the return of the stricture, and had to again go through the whole process of dilatation. His urine was acid, with a slight trace of albumen and some mucus deposit. From this condition of urine it was probable that he had some slight degree of sympathetic cystitis.

Prostatic Cases.—With regard to enlarged prostate it is generally acknowledged that many men have considerable enlargement of their prostate without having any symptoms, or, in fact, without being in the least aware of their condition, the reason probably being that the prostate in these cases in its growth does not in any way interfere with the mechanical process of micturition. A specimen showed this condition very well. It was taken from a man who had died from a rapid form of cerebral affection. The preparation showed a peculiar bifid prostate, the gland being almost split in two, with a groove between the two hypertrophied lobes; and yet this patient had never shown the slightest symptom of prostatic trouble. Another case of interest in this direction, was that of a gentleman who for many years had passed an instrument regularly for fear of getting prostatic obstruction; after death, there was a very well formed groove along the prostatic urethra from the constant usage of the bougie. In all cases of early symptoms of prostatic trouble the patient should pass a bougie regularly himself. This much we realize, there is no process which prevents the hypertrophy of the prostate, but we can by early regular treatment prevent this from ever leading to serious symptoms. A question often asked was, when should a man begin the use of a catheter, or in fact begin catheter life? The answer was, when he cannot do without it, that is to say, when for instance he has to get up several times at night to pass water, and then is unable to completely empty his bladder, then it is time to commence using an instrument. A caution is here necessary: if a bladder has been accustomed to hold continually some six or eight ounces of urine, a portion of this only should be removed the first time an instrument is passed, for fear of setting up catheter fever.

The third case shown was that of a man with an urethral stricture and also an enlarged prostate. The fourth case was a man with incontinence who had a very large prostate producing frequency of micturition; the urine was acid and contained some mucus, otherwise it was normal. An enlarged prostate is liable to temporary attacks of inflammation and so incontinence may be produced. In examining for enlarged prostate it is better to examine with the patient standing up and leaning well forward over the end of a couch or other suitable article.

